# Stick at It, Fellow Veterinarians

**Published:** 1897-06

**Authors:** 


### EDITORIAL.

### STICK AT IT, FELLOW VETERINARIANS.

Illinois veterinarians, and those of Chicago in particular, have much cause to feel aggrieved at the actions of the chief executive of the State in appointing non-graduates to fill official positions in the veterinary service of the State and city. We have no disposition to discuss the merits or demerits of those appointed to these places; we have only to deal with the need in these times of every State and community to secure the best equipped and broadest-gauged men to be found in their respective communities. The rapid advancement of veterinary science, its place among the learned professions of the day, its daily increasing importance in the field of comparative and sanitary medicine, bring it, in every aspect of its work, in closer relationship to man, leaving no good reason for any other selection than the best educated and the most advanced members in the profession to properly fill such important positions. Surely none but those best equipped with the best college education, combined with practical experience, should be called to such places, and the profession is right in protesting and condemning appointments made on any other grounds. It should continue to protest, loudly and emphatically, and thus educate public sentiment to the importance of filling these places with educated veterinarians. Let the people know that the relations between man and the lower animals enter into the whole question of a pure food-supply, thus affecting in a very important aspect the welfare of both. Much is known already in these matters, but it is only a tithe of what there is to be studied and determined to guard against the dangers, suffering, and losses that are daily falling upon our people, and only the most advanced thinkers and best educated can plan for each State and city to do its part in this great work. Strengthen your numbers in this protest; reform your lines and agitate the matter until your protest is heard and answered. Your organizations are the channels for this work; the profession will sustain you, and journalism will aid you in this worthy fight for advanced veterinary education.

